# Magnifying Endoscopic Findings Can Predict Clinical Outcome during Long-Term Follow-Up of More Than 12 Months in Patients with Ulcerative Colitis

**DOI:** 10.1155/2013/671576

**Published:** 2013-10-02

**Authors:** Hajime Isomoto, Ryohei Uehara, Tomayoshi Hayashi, Junya Shiota, Kayoko Matsushima, Chun Chuan Chen, Fuminao Takeshima, Toshiyuki Nakayama, Kazuhiko Nakao

**Affiliations:** ^1^Department of Gastroenterology and Hepatology, Nagasaki University Hospital, 1-7-1 Sakamoto, Nagasaki 852-8102, Japan; ^2^Department of Pathology, Nagasaki University Hospital, Nagasaki 852-8102, Japan

## Abstract

*Background and Aims*. To explore the association of magnifying endoscopic (ME) findings with histopathology and relapse in ulcerative colitis (UC). *Methods*. Forty-six patients with UC underwent ME with narrow band imaging (NBI) and crystal violet staining and were followed for more than 12 months. ME findings with vital staining were classified into ME-A, regular arrangement of round to oval pits; ME-B, irregular arrangement with/without enlarged spaces between even pits; ME-C, irregular pits in size and shape with more irregular arrangement of pits; and ME-D, disrupted or disappeared pits. NBI-guided ME features of microvascular pattern (MVP) were divided into the MVP-regular and MVP-irregular type. *Results*. There were 5, 24, 10, and 7 cases of ME-A, ME-B, ME-C, and ME-D grade, respectively, while there were 21 and 25 of MVP-regular and MVP-irregular type, respectively. ME classifications were significantly associated with Matts endoscopic grade. ME classifications and MVP types were significantly associated with each pathognomonic microscopic feature of severe mucosal inflammation, crypt abscess, and goblet cell depletion. There were significant differences in the percentages of remission among ME classifications and between MVP types. *Conclusion*. ME findings can be predictive of relapse in UC and reliable for *in vivo* histopathological assessment.

## 1. Introduction

Ulcerative colitis (UC) is a chronic inflammatory bowel disorder characterized by diffuse mucosal inflammation of the colorectum with exacerbations and remissions. The diagnosis of UC is obtained by incorporating clinical, laboratory, radiological, endoscopic, and histopathological findings [1]. Assessment of UC activity via conventional colonoscopy is essential to determine the extent and severity of the disease to optimize therapeutic strategy [1]. Higher severity of endoscopic lesions in UC predicts more aggressive clinical courses and increased rates of surgery [2]. Nevertheless, there are discrepancies between colonoscopic and histopathological findings in patients with clinically inactive UC. Even when routine colonoscopy suggests remission with the normal-appearing mucosa, that is, a common, standardized diagnosis of anatomic remission in accordance with the clinical feature, microscopic inflammatory abnormalities may persist and relapse may occur sooner or later. In the clinical setting of inactive UC, therefore, the disease relapse is difficult to be predicted by routine colonoscopy alone [1, 2].

Magnifying endoscopy (ME) with vital staining is useful for diagnosing colorectal neoplasms by means of precise observation of surface pit patterns [3]. Again, narrow band imaging (NBI) is an innovative optical technology to enhance hemoglobin-rich areas such as blood vessels [4], and, coupled with NBI, ME depicts the superficial microvascular architectures in the neoplastic lesions [5]. Recent studies have showed that the ME features of colorectal mucosa in UC patients may be associated with histopathological activity of UC and enable the prediction of relapse even in the inactive disease [6, 7]. However, the reported observation periods for the relapse assessment were relatively short-term within 12 months in earlier studies. Herein, we have proposed ME stratification based on alterations in the surface pit patterns or microvascular architectures in the rectal mucosa of patients with inactive UC. The current study suggests that the ME findings with vital staining and NBI can predict clinical outcome of UC during longer-term follow-up. 

## 2. Patients and Methods

Between October 2010 and November 2011, we recruited consecutive patients previously diagnosed with UC who visited the outpatient clinic of Nagasaki University Hospital for colonoscopic surveillance. Exclusion criteria were defined as follows: inability to provide written informed consent, severe coagulopathy, cirrhosis, impaired renal dysfunction, pregnancy or breast feeding, active gastrointestinal bleeding, or known allergy to methylene blue or crystal violet. UC patients in the active stage with aggressive endoscopic disease were also excluded from the study. The diagnosis of UC was based on established clinical, radiologic, endoscopic, and histopathological findings. The study was approved by the Nagasaki University Ethics Committee and was conducted in accordance with the Helsinki Declaration.

**Figure 1:**
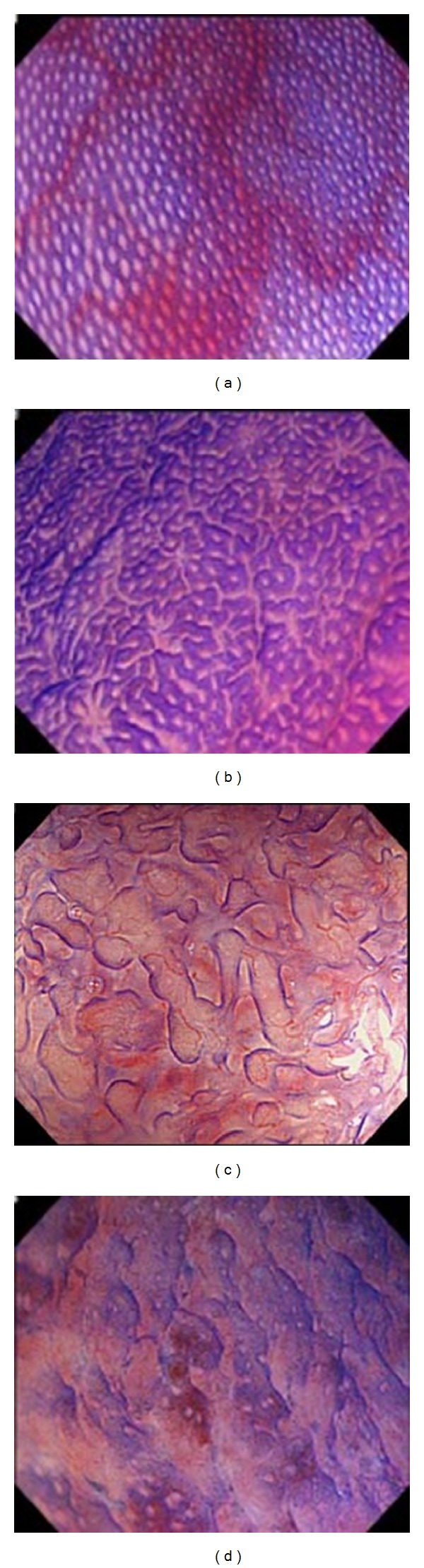
Magnifying endoscopic (ME) findings following crystal violet staining were classified into 4 grades as follows: ME-A, regular arrangement of normal-appearing round to oval pits (a); ME-B, irregular arrangement with/without enlarged spaces between the even pits (b); ME-C, irregular pits in size and shape with more irregular arrangement of pits compared to ME-B (c); and ME-D, disrupted or disappeared pits (d).

**Figure 2:**
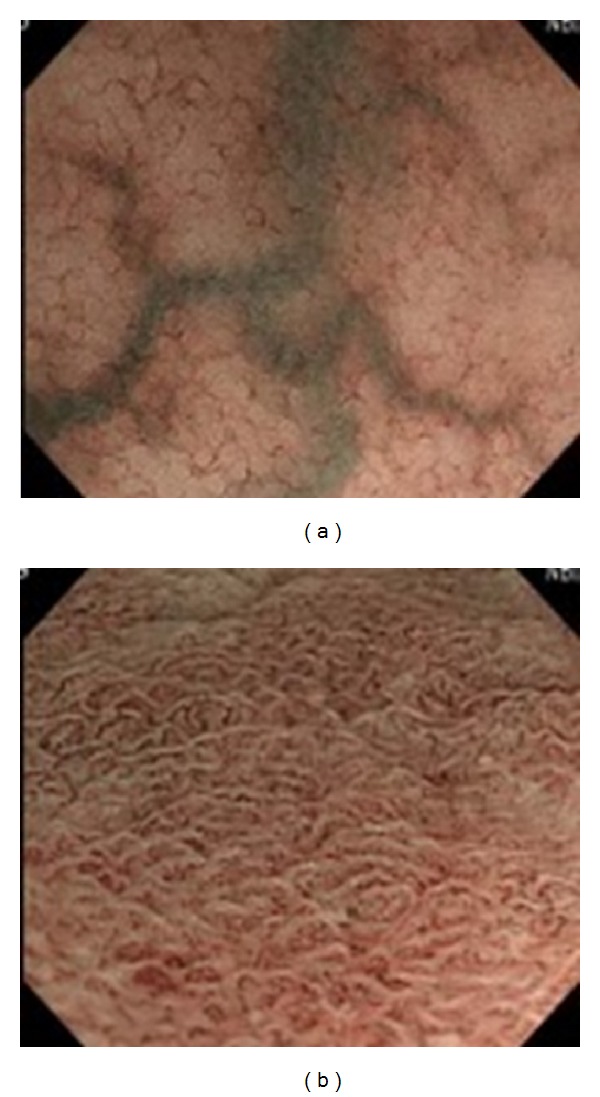
Narrow band imaging- (NBI-) guided magnifying endoscopic findings of microvascular pattern (MVP) were divided into either MVP regularly arranged in a honeycomb-like structure (MVP-regular (a)) or MVP with irregular, tortuous structure (MVP-irregular (b)).

The endoscopic system included a light source (CLV-260SL; Olympus Optical Co., Tokyo, Japan), a processor (CV-260SL; Olympus), and a colonoscope (PCF-Q260AZI; Olympus). After endoscopic insertion, luminal lavage was conducted with water including dimethicone and pronase. Following standard observation, ME following vital staining including 0.05% crystal violet was conducted in the rectal mucosa. Then, ME findings were classified into 4 types as follows: ME-A grade (ME-A), regular arrangement of normal-appearing round to oval pits; ME-B, irregular arrangement of round to oval pits with/without enlarged spaces between the even crypts; ME-C, irregular pits in size and shape with more irregular arrangement of the pits compared to ME-B; and ME-D, destruction and disappearance of pits (Figure 1). When the ME findings of more than one grade were present in the same case, the higher grade was recorded. NBI-guided magnifying observation was also conducted to evaluate alterations in the mucosal microvascular pattern (MVP) which was divided into either MVP regularly arranged in a honeycomb-like structure (MVP-regular) or MVP with irregular, tortuous structure (MVP-irregular) in accordance with the previous classification [8] (Figure 2). In the end, targeted biopsy sampling was performed for histopathological evaluation.

Biopsy specimens were fixed with 10% formalin and embedded in paraffin, and sections were stained with hematoxylin and eosin. The pathologist, who was blinded to clinical and endoscopic information, evaluated the slices for histopathological assessment of inflammation in UC, with special reference to the severity of mucosal inflammatory infiltration and the presence of crypt abscesses and goblet cell depletion. 

The ME findings were compared to clinicopathological characteristics, including disease activity and extension and clinical course. All patients were followed monthly or bimonthly for more than 12 months. Relapse was investigated [9].

Statistical analyses were performed using Fisher's exact test, the *χ* ^2^ test, Student's *t*-test, the Mann-Whitney *U* test, and the Kruskal-Wallis test as appropriate. Comparison of the percentage of patients in remission on the basis of endocytoscopic classification was made using the Kaplan-Meier method and analyzed by the log-rank test. A *P* value < 0.05 was accepted as statistically significant. 

## 3. Results

Forty-six patients with UC were enrolled in this study and underwent ME. According to ME stratification, there were 5 cases of the ME-A grade, 24 cases of the ME-B, 10 cases of the ME-C, and 7 cases of the ME-D. On the other hand, they were divided into the MVP-regular type (*n* = 21) and MVP-irregular type (*n* = 25). The cases of MVP-regular type belonged to the ME-A (*n* = 5), ME-B (*n* = 14), and ME-C (*n* = 2) group, while, in the MVP-irregular type, there were 10, 8, and 7 cases of the ME-B, ME-C, and ME-D grade, respectively.

The patients consisted of 30 men and 16 women: there were no significant differences in gender among the ME classifications, while the MVP-R type was male predominant (17 men out of 21 cases) compared with the MVP-IR type (13 men out of 25 cases) (*P* < 0.05). The mean age at entry was 34.2 years (range, 14–77 years). The mean age of the ME-A cases was higher with 54.2 years compared with 32.9, 28.5, and 32.7 years in the ME-B, ME-C, and ME-D cases, respectively, reaching statistical significance for each (*P* < 0.05). The mean ages were comparable irrespective of the MVP types. The duration of the disease was 14 years (range, 0–29 years) without statistical differences among the ME classifications and between the MVP types. The diagnoses included 17 pancolitis, 10 left-sided colitis, and 19 proctitis type without significant differences in the distribution among the ME classifications and between the MVP types. There were 41 UC patients experiencing relapse-remission and 5 patients suffering the first attack of UC without significant differences in the distribution among the ME classifications and between the MVP types.

We summarized the association of different therapy regimens with Matts grades, ME classification, and MVP patterns in Table 1. UC patients, who were prescribed mesalazine plus azathioprine and hence are not in so quiescent course of the disease, tended to have higher ME classes and more irregular MVP pattern compared with those treated with 5-aminosalicylic acid alone (mesalazine or salazosulfapyridine) despite statistical insignificance. Again, 6 patients who did not receive any specific therapies due to the quiescent disease had the diverse ME classification and MVP patterns, suggesting the discrepancy between such magnified endoscopic findings and clinical activity. The correlations between the different therapies, Matts grades, ME classification, and MVP patterns appeared poor. As a rule, kinds and dosages of the therapeutic drugs for UC were unchanged during the study period.

Fifteen of the cases were classified as Matts 1, while 31 of the cases were classified as Matts-2 endoscopic grade of UC. As for the relationship between the Matts endoscopic grade and ME classifications, each ME-A case was categorized into Matts 1 grade, while the ME-B cases were divided into Matts-1 (*n* = 10) and Matts-2 (*n* = 14) grade. All the ME-C and ME-D cases were categorized into Matts-2 grade. Thus, there were 14 ME-B, 10 ME-C, and 7 ME-D cases in the Matts-2 grade group. There was a significant association of the ME classifications with Matts endoscopic grades (*P* < 0.0005). As for the relationship between the MVP types and Matts endoscopic grade, the MVP-regular cases were divided into Matts-1 (*n* = 14) and Matts-2 (*n* = 7) grade, while the NBI-irregular cases were divided into Matts-1 (*n* = 1) and Matts-2 (*n* = 24) grade. Thus, there were 7 MVP-regular and 24 MVP-irregular cases in Matts 2. We performed NBI magnification followed by chromomagnifying observation with crystal violet as gently and quickly as possible to avoid excessive mucus exudate as well as contact bleeding within the granular mucosa of UC cases in the Matts-2 grade. Nevertheless, Table 2 summarized that correlations between Matts 2, ME classification, MVP type, and therapy were weak, indicating the diversity in microsurface and microvascular patterns in UC cases of Matts-2 class.

Table 3 shows the correlations between the ME classifications and the histopathological findings specific for the disease. In the 4 cases, the specimens were not evaluable and excluded in the analyses. The differences in the presence of severe mucosal inflammation, crypt abscesses, and goblet cell depletion among the ME classifications reached statistical significance for each.  Table 4 shows the correlations between the MVP types and the pathognomonic histopathological findings of UC. The differences in the presence of severe mucosal inflammation, crypt abscesses, and goblet cell depletion between the MVP types reached statistical significance for each.

**Figure 3:**
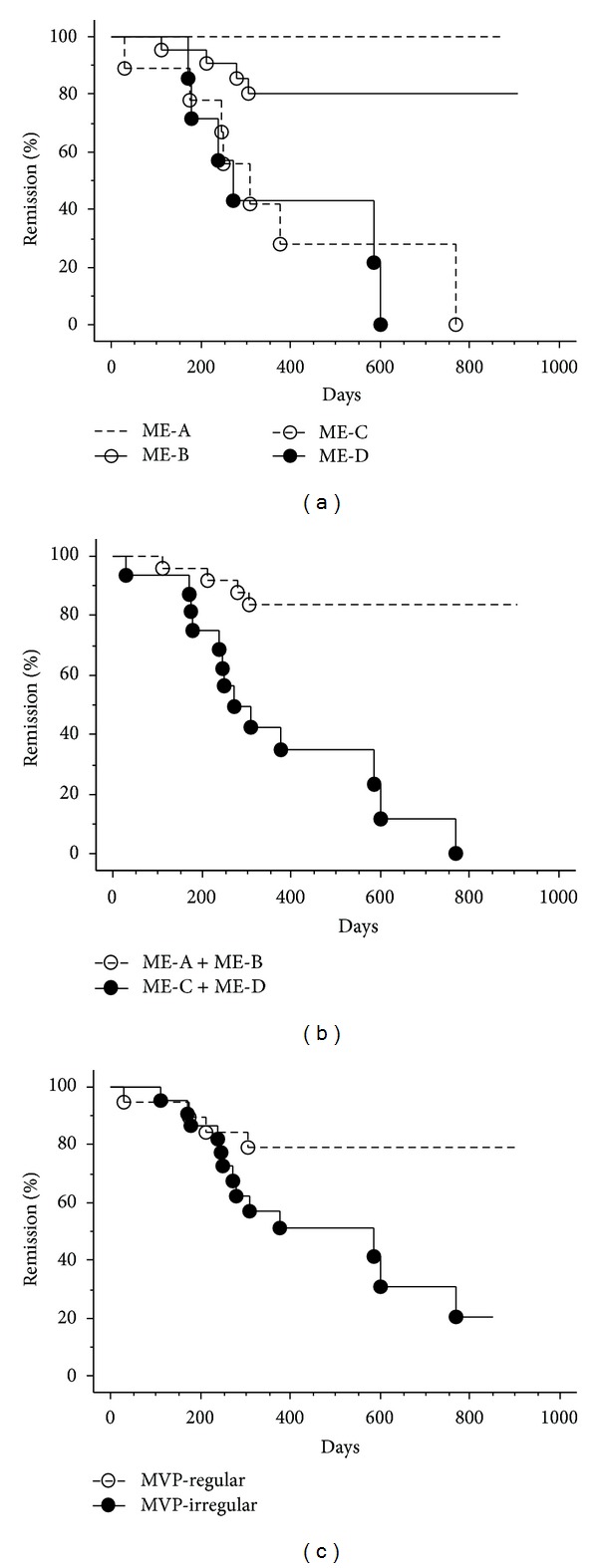
Comparison of the percentage of patients in remission on the basis of the ME classifications with vital staining or NBI using the Kaplan-Meier method. There are significant differences in the percentages of the remission of UC among the ME classifications (a), between ME-A plus ME-B group and the ME-C plus ME-D group (b), and between the MVP-regular and MVP-irregular type (c).

As for the long-term subsequent outcome following the study inclusion, there were significant differences in the percentages of UC remission among the ME classifications (*P* < 0.005, Figure 3(a)). When combined, there was a rather significant difference in the percentages of the remission between the ME-A plus ME-B group and the ME-C and ME-D group (*P* < 0.0001, Figure 3(b)). There was a significant difference in the percentages of the remission between the MVP types (*P* < 0.05, Figure 3(c)). In turn, there were significant differences in the disease relapse incidences during the past 12 months among the ME classifications or between the MVP types (Table 5). None had surgical intervention prior to the study entry or during the follow-up period. 

## 4. Discussion

We documented the 4-grading system using ME findings with vital staining in the rectal mucosa of UC patients. The ME classifications were significantly associated with the UC-pathognomonic microscopic features including severe mucosal inflammation, crypt abscess, and goblet cell depletion. The final diagnosis and evaluation of inflammation activity in UC as well as Crohn's disease are still predominantly based on histopathological analysis [10, 11]. Nevertheless, the preparation of tissue sections and ensuing histopathological examination usually take considerable time. Due to sampling errors, histopathological assessment is not representative of the entire inflamed area. In addition, the possible risk of physical bleeding may give reason for use of alternative methods for nondestructive *in vivo* imaging modalities [11]. In line with our results, several studies have demonstrated that the ME findings significantly correlate with the histological grades of UC [12, 13]. Mild magnifying endoscopic finding with lower grade of their classification corresponding to our ME-B type showed moderate or higher degree of histological mucosal inflammation in the earlier studies [6]. Thus, there could be a distinct difference in microscopic pathognomonic features even between the inactive UC mucosa classified into ME-A and ME-B. In addition, the highly accurate optical biopsy via the ME imaging may limit the number of biopsies required with substantial cost savings for pathology services [14]. More recently, endocytoscopy and confocal endomicroscopy provide real-time microscopic images at no less than 100-fold magnification during ongoing endoscopy [15, 16]. However, such ultramagnification endoscopic technology is currently unavailable in the clinical practice settings.

Fujiya et al. classified the ME findings following spraying a contrast dye, indigo carmine onto the mucosa into 5 grades (regular arranged crypt openings, villous-like, minute defects of epithelium, small yellowish spots, and coral reef-like appearance) in the severely affected mucosa by UC varying the Matts-1 to Matts-4 endoscopic grade [12]. In the similar way using the indigo carmine spray, there was a report on the modified 6-grading system by adding the ME appearance of polypoid mucosal tags [13]. A recent study claimed the 3-grading system of ME findings with the indigo carmine spray consisting of magnify-regular, magnify-irregular, and magnify-defect in patients with the inactive disease [7]. On the other hand, Nishio et al. classified the ME features after staining with an absorptive dye, methylene blue into 4 grades in the rectal mucosa of inactive UC patients [6], similar to the present study where crystal violet was employed as the absorptive dye. The contrast staining lasts for only a few minutes and disappears owing to dilution or exudates. On the other hand, the absorptive dyes yield stable stain even into the cellular nuclei allowing sufficient and avid examinations particularly in the case of ME observation [17]. Then, fine surface patterns of neoplastic as well as nonneoplastic lesions can be readily identified providing rather clear magnification images. Nevertheless, the ME using such absorptive staining has potential limitations related to the additional processes of repeated washing, staining, and imaging.

NBI can clearly enhance and visualize the microvascular structures of the mucosal layer as a result of the differential optical absorption of light by hemoglobin [3]. The resultant images look like chromoendoscopy without dye [17]. Then, NBI in combination with ME is useful to characterize the colorectal neoplastic lesions [5]. Esaki et al. reported that NBI observation was of value for the precise assessment of histological severity of the mucosa affected by UC. The NBI-guided ME finding of either the MVP-regular or MVP-irregular in the inactive mucosa showed close association with histopathological findings including crypt distortion, basal plasmacytosis, and goblet cell depletion [8] as seen in the present study. In addition, we have found the MVP-irregular type was associated with the presence of crypt abscess, the pathognomonic microscopic appearance of UC. 

The present study has demonstrated that ME stratification can predict disease relapse in UC patients in line with prior studies. Watanabe et al. reported that the magnify-regular group, as mentioned above, had the significant lower relapse rate within 12 months compared to the magnify-irregular and magnify-defect group [7]. In the other study, UC relapse during 12 months of follow-up was found to be increased with worsening grade by the 4 ME classifications [6]. Our series of UC were followed up more than at least 12 months without exception, and the Kaplan-Meier estimate has shown that the 4-grade stratification can be useful to predict the clinical outcome for longer period. In particular, the ME-C and ME-D group had relapse exclusively (Figure 3(b)) despite the quiescent disease at the entry. Of clinical note, the current data indicate that most ME-A and ME-B cases can maintain the long-lasting remission of UC, given the 5-aminosalicylate alone. On the other hand, the MVP types could also predict the probability of UC relapse (Figure 3(c)). To our knowledge, the present study for the first time assessed the prediction of disease relapse in UC patients via the NBI-guided ME approach. Current data from the present study provides insights to enable the design of further prospective clinical trials to assess whether the ME findings based on crypt or microvessel architectures can predict clinical outcome. The ME classifications as well as the MVP types at the entry were associated with the 12-month preceding episode of UC relapse as shown in Table 3 and possibly could reflect the degree and sustention of mucosal healing, eventually linking to the disease remission. 

In conclusion, ME is a reliable method for assessing the microscopic inflammatory features of UC culminating in the provision of an *in vivo* histopathological evaluation during ongoing endoscopic examination. ME stratification based on crypt microstructures or MVP in UC patients can predict longer-term disease outcome.

## Figures and Tables

**Table 1:** Summary of relationship between ulcerative colitis treatment, Matts endoscopic grade, magnifying endoscopic (ME) classifications following crystal violet staining, and microvascular pattern (MVP).

Therapy	Matts grade		ME classification				MVP type	
	Matts 1	Matts 2	ME-A (*n* = 5)	ME-B (*n* = 24)	ME-C (*n* = 10)	ME-D (*n* = 7)	MVP-R (*n* = 21)	MVP-IR (*n* = 25)
Mesalazine (*n* = 29)	7	22	2	15	9	3	11	18
Salazosulfapyridine (*n* = 5)	4	1	2	3	0	0	4	1
Azathioprine (*n* = 1)	1	0	0	1	0	0	1	0
Azathioprine + mesalazine (*n* = 5)	1	4	0	2	0	3	1	4
None (*n* = 6)	2	4	1	3	1	1	4	2

ME-A: regular arrangement of normal-appearing round to oval pits; ME-B: irregular arrangement with/without enlarged spaces between the even pits; ME-C: irregular pits in size and shape with more irregular arrangement of pits compared to ME-B; and ME-D: disrupted or disappeared pits. MVP-regular: regularly arranged in a honeycomb-like structure; MVP-irregular: MVP with irregular, tortuous structure.

**Table 2:** Summary of relationship between ulcerative colitis treatment, magnifying endoscopic (ME) classifications following crystal violet staining, and microvascular pattern (MVP) in cases of Matts-2 endoscopic grade.

Therapy	ME classification				MVP type	
	ME-A (*n* = 0)	ME-B (*n* = 14)	ME-C (*n* = 10)	ME-D (*n* = 7)	MVP-R (*n* = 7)	MVP-IR (*n* = 24)
Mesalazine (*n* = 22)	0	10	9	3	5	17
Salazosulfapyridine (*n* = 1)	0	1	0	0	0	1
Azathioprine + mesalazine (*n* = 4)	0	1	0	3	0	4
None (*n* = 4)	0	2	1	1	2	2

ME-A: regular arrangement of normal-appearing round to oval pits; ME-B: irregular arrangement with/without enlarged spaces between the even pits; ME-C: irregular pits in size and shape with more irregular arrangement of pits compared to ME-B; and ME-D: disrupted or disappeared pits. MVP-regular: regularly arranged in a honeycomb-like structure; MVP-irregular: MVP with irregular, tortuous structure.

**Table 3:** Magnifying endoscopic (ME) classifications following crystal violet staining and pathognomonic histological features of ulcerative colitis.

Magnifying endoscopic (ME) classification	Severe mucosal inflammation		Crypt abscess		Goblet cell depletion		Total case
	Present	Absent	Present	Absent	Present	Absent	
ME-A	0	5	0	5	0	5	5
ME-B	8	14	8	14	12	10	22
ME-C	9	0	7	2	8	1	9
ME-D	5	1	5	1	6	0	6
*P* value	<0.0005		<0.01		<0.0005		42

ME-A: regular arrangement of normal-appearing round to oval pits; ME-B: irregular arrangement with/without enlarged spaces between the even pits; ME-C: irregular pits in size and shape with more irregular arrangement of pits compared to ME-B; and ME-D: disrupted or disappeared pits.

**Table 4:** Narrow band imaging-guided magnifying endoscopic findings of microvascular pattern (MVP) and pathognomonic histological features of ulcerative colitis.

Microvascular pattern (MVP) type	Severe mucosal inflammation		Crypt abscess		Goblet cell depletion		Total case
	Present	Absent	Present	Absent	Present	Absent	
MVP-regular	3	16	3	16	4	15	19
MVP-irregular	19	4	17	6	22	1	23
*P* value	<0.0001		<0.0005		<0.0001		42

MVP-regular: regularly arranged in a honeycomb-like structure; MVP-irregular: MVP with irregular, tortuous structure.

**Table 5:** The episode of disease relapse during the past 12 months among the ME (magnifying endoscopic) classifications and between the MVP (microvascular pattern) types of patients with ulcerative colitis.

	ME classification				MVP type	
	ME-A (*n* = 5)	ME-B (*n* = 24)	ME-C (*n* = 10)	ME-D (*n* = 7)	MVP-R (*n* = 21)	MVP-IR (*n* = 25)
Relapse during the past 12 months						
Present	0	4	7	6	4	13
Absent	4	17	2	1	15	9
First attacking	1	3	1	0	2	3
*P* value	*P* < 0.005				*P* < 0.05	

ME-A: regular arrangement of normal-appearing round to oval pits; ME-B: irregular arrangement with/without enlarged spaces between the even pits; ME-C: irregular pits in size and shape with more irregular arrangement of pits compared to ME-B; and ME-D: disrupted or disappeared pits.

MVP-R: regular, regularly arranged in a honeycomb-like structure; MVP-IR: irregular, MVP with irregular, tortuous structure.
